# The (Self) Elect, Etc

**Published:** 1886-03

**Authors:** 


### THE (SELF) ELECT.

"We thank Thee, oh, Lord, that we are not as other (Doctors) are." Drs. Delafield, *President;* Pepper, Loomis, Draper and Tyson (Secretary) of Pennsylvania and New York; and Welch, of Baltimore, and Howard, of Canada, and Minot, of Boston, have constituted themselves "a new National Medical Association" (to take the place of the present A M. A.—whose funeral at St. Louis is now being plotted.) They graciously announce that they will receive ninety-two more, of their own selection, into the inner temple; then shut the door. Ah! but it's to be fine!

This is the most beautiful illustration we have ever seen of Josh Billings' story of the stump of the tail wagging the dog! says Dr. Merryman. This is an association *as is* an association, and like unto it there is nothing in the heavens above, nor in the waters beneath—no, not anywhere! Selah!

---

The *New York Sanitarian* pays us the following handsome compliment:

"DANIEL'S TEXAS MEDICAL JOURNAL is one of the most progressive and cogent periodicals on our exchange list,—always fresh in both matter and manner."

---

The *Kansas City Medical Index* says: "Dr. Daniel has made a new departure in medical journalism, meeting with unprecedented success. His journal is the *most intensely original* periodical that reaches our table."

---

### A GREAT ADVANCE IN SURGERY.

The *New York Medical Journal* gives the report of a case where amputation of the thigh was successfully performed without chloroform, ether or other general anæsthetic, cocaine alone being used—locally—after the method of Dr. Corning; by arresting the circulation with a tourniquet, and thus prolonging the anæsthetic effects of the drug. Those of our readers who have not done so should read Dr. Corning's paper on prolonging the effect of cocaine.

### Abbreviating Prescriptions.

An old practitioner. a friend of ours, who invariably writes his prescriptions in English. and in full at that, gives as a reason that on one occasion in a prescription he wrote, "Aqua. font.," and the druggist, reading it "aquafort.," actually put up sulphuric acid—"aquafortis!"

---

### A GOOD SHOWING..

The report of the Secretary at the annual meeting of the Directors and Faculty of the New York Polyclinic, held at the College Building, on January 28, 1886, showed an attendance upon the clinic in that institution, since the opening on November 7, 1882, of 709 physicians.

The ratio of attendance upon the various departments is shown in the following list of tickets sold since November, 1882, up to January 28, 1886: Gynecology, 461; Surgery, 412; Medicine, 313; throat, nose and ear, 300; diseases of children, 273; diseases of eye, 250; diseases of skin, 234; diseases of mind and nervous system, 207; Physiological Chemistry, 173; Obstetrics, 163; Pathology (laboratory only recently opened), 15. Total, 2801. The attendance for the present session is in excess of any previous term.

---

Dr. E. J. Beall, of Fort Worth, requests us to say that he will leave for New York about the 20th inst., for "a six weeks' course of study and observation in that great medical center;" and that, having made such trips, and being perfectly familiar with everything incident to such a trip, and moreover desiring company, he will undertake to chaperone a party of medical gentlemen, bent on the same purposes. He can give them all desirable information as to expenses, where best to go on arrival, etc.; what to see and how to see it, etc.; and to this end he desires to open correspondence with such physicians as contemplate the trip.

---

Dr. F. T. Paine, late of Comanche, Texas, who, it will be remembered, has contributed several papers to this journal on electric treatment of disease, and who is the author of the paper on *Salix Niger* in the transactions of the Texas State Association, which has attracted such wide attention, we are pleased to say, has removed to Austin, and opened an office for practice. His practice is limited to treatment of diseases of nervous system and generative organs in male and female, by electricity.

